# Auswirkungen der Digitalisierung auf das Weiterbildungsangebot für gering Qualifizierte. Eine datenbankbasierte Angebotsanalyse für Rheinland-Pfalz

**DOI:** 10.1007/s40955-023-00236-y

**Published:** 2023-03-08

**Authors:** Sophie Lacher, Matthias Rohs

**Affiliations:** 1 Rheinland-Pfälzische Technische Universität Kaiserslautern-Landau, Campus Kaiserslautern, Kaiserslautern, Deutschland; 2 Rheinland-Pfälzische Technische Universität Kaiserslautern-Landau, Campus Kaiserslautern, Kaiserslautern, Deutschland

**Keywords:** Weiterbildung, Erwachsenenbildung, Digitalisierung, Weiterbildungsdatenbank, Geringqualifizierte, Continuing education, Adult education, Digitalization, Database, Low-skilled

## Abstract

Im Zusammenhang mit der Digitalisierung im Bildungsbereich werden vielfach auch Risiken einer damit verbundenen Ungleichheit diskutiert. In diesem Beitrag werden aus dieser Perspektive die Auswirkungen der Digitalisierung der Weiterbildung für „gering Qualifizierte“ im Bereich der Computer‑/IT-Grundlagen dargestellt. Der Datenzugang erfolgt über bisher in der Angebotsforschung kaum genutzte Weiterbildungsdatenbanken. Die Ergebnisse zeigen, dass den besonderen Anforderungen der Zielgruppe an digitalisierte Weiterbildungsangebote durch entsprechende Maßnahmen entgegengekommen werden kann, gleichzeitig aber die Heterogenität der Zielgruppe nur unzureichend berücksichtigt wird.

## Abstract (en)

In the context of digitization in education, the risks of inequality are often discussed. From this perspective, the article examines the effects of the digitalization of continuing education for the “low-skilled” in the area of computer/IT basics. The data is accessed via continuing education databases, which have hardly been used in offer research to date. The results show that the special requirements of the target group for digitalised continuing education can be met by appropriate programs, but at the same time the heterogeneity of the target group is not sufficiently considered.

## Einleitung

Durch das Verbot von Präsenzveranstaltungen während der Corona-Pandemie ist es zu einem Einbruch des Weiterbildungsangebots gekommen (Christ et al. 2021). Gleichzeitig führte die mit den pandemischen Maßnahmen einhergehende Digitalisierung des Weiterbildungsangebots zu einer Zunahme der Unterschiede in der Weiterbildungsbeteiligung zwischen niedriger und höher qualifizierten Erwachsenen im Vergleich zur Zeit vor der Pandemie (Ehlert et al. 2021). Vor diesem Hintergrund stellt sich die Frage nach den Zusammenhängen zwischen der Digitalisierung von Weiterbildungsangeboten und der zurückgehenden Weiterbildungsbeteiligung gering Qualifizierter. Mit der Gruppe der gering Qualifizierten, welche in Deutschland ca. 5 Mio. Menschen umfasst (Martin et al. 2015, S. 57), wird in der hier vorgestellten Untersuchung ein Personenkreis in den Blick genommen, welcher sich grundsätzlich in nur sehr geringen Umfang an Weiterbildung beteiligt (ebd., S. 59) und gleichzeitig durch die Anforderungen der Digitalisierung von Arbeit sowie arbeitsmarkbezogenen Krisen wie während der Pandemie von zunehmender Segregation betroffen ist.

Von spezifischem Interesse ist in diesem Zusammenhang, wie das digitale Weiterbildungsangebot für gering Qualifizierte im Kontext einer allgemein forcierten Digitalisierung im Weiterbildungsbereich (Christ et al. 2020) gestaltet ist. Dazu wird der aktuelle Status der Angebotsgestaltung in den Blick genommen, in welchem sich die Folgen der pandemiebedingten Digitalisierung bereits deutlich zeigen, ohne dass davon ausgegangen wird, dass dieser Prozess bereits abgeschlossen ist. Ziel ist es aufzuzeigen, wie digitalisierte Weiterbildungen für gering Qualifizierte didaktisch gestaltet sind und wie spezifische Zugangsbarrieren für die Zielgruppe in diesem Bereich berücksichtigt werden, um mögliche Exklusionsmechanismen zu vermeiden, bzw. wie durch die Gestaltung der Angebote die besonderen Voraussetzungen der Zielgruppe Beachtung finden. Daraus resultieren die drei leitenden Forschungsfragen:

1. Welche inhaltlichen und didaktischen Merkmale weisen Weiterbildungsangebote mit Bezug zur Digitalisierung für gering Qualifizierte auf?
2. Was wird für eine Teilnahme vorausgesetzt (z. B. technische Ausstattung, medienbezogene Kompetenzen)?
3. Wie werden die fehlenden oder unzureichenden Voraussetzungen der Zielgruppe für die Weiterbildungsangebote kompensiert?

Um eine möglichst große Varianz von Anbietern und Angeboten zu berücksichtigen, wird als Datenzugang die Weiterbildungsdatenbank KURSNET^1^ der Bundesagentur für Arbeit als „Deutschlands größte Onlineplattform für Weiterbildungen“ (BMAS und BMBF 2019, S. 7) genutzt.

## Gering Qualifizierte im Kontext der Digitalisierung

Gering Qualifizierte zeichnen sich durch eine große Heterogenität aus, was sich in unterschiedlichen Akzentsetzungen in bestehenden Definitionen ausdrückt (z. B. Bertelsmann Stiftung 2018, S. 10; CEDEFOP 2018, S. 2). Zu diesen Definitionen wird kritisch angemerkt, dass sie sich grundsätzlich auf das Bildungsniveau beziehen (CEDEFOP 2018, S. 2) und die Bezeichnung „gering Qualifizierte“ einen stigmatisierenden Charakter hat (Krenn 2012, S. 130). Im Rahmen des Grundlagenforschungsprojekts FORWARD^2^ wurden die unterschiedlichen Kriterien zur Beschreibung der Zielgruppe analysiert (Lacher et al. 2022). Als Ergebnis entstand die nachfolgende Definition von „gering Qualifizierten“, welche die Heterogenität der Perspektiven berücksichtigt und eine relative Positionierung vornimmt:

„Geringqualifizierte sind erwachsene Menschen, welche in Bezug auf konkrete berufliche Tätigkeitsbereiche zu einem bestimmten Zeitpunkt nicht oder nicht mehr über die als notwendig erachteten Kompetenzen verfügen. Die Bewertung *geringqualifiziert* erfolgt dabei zum einen in Bezug auf das national durchschnittliche formale Bildungsniveau und zum anderen auf das Vorhandensein berufsqualifizierende Abschlüsse [sic] bzw. non-formal und informell erworbener Kompetenzen sowie deren zeitliche und inhaltliche Passung.“ (Lacher et al. 2022, S. 11; Hervorh. i. O.).

Betrachtet man gering Qualifizierte ohne formalen Berufsabschluss, gilt für diese, dass sie im Durchschnitt schlechtere Chancen auf dem Arbeitsmarkt haben als Personen mit Berufsabschluss (Flake et al. 2017). Einerseits gestaltet es sich für diese Personengruppe schwer, überhaupt Zugang zum Arbeitsmarkt zu finden, anderseits sind ihre Tätigkeiten vielfach durch instabile Erwerbsverläufe geprägt sowie mit einem erhöhten Risiko verbunden, im Niedriglohnsektor tätig zu sein oder einer prekären Beschäftigung nachzugehen (Dietrich et al. 2019). Gleichzeitig hat die Digitalisierung auch die Arbeitsplätze von Personen mit niedrigem Ausbildungsniveau erreicht (Bömer et al. 2020). In der Folge werden mittlerweile bei 62 % aller Stellenanzeigen für gering Qualifizierte digitale Grundkompetenzen wie das Bedienen eines Computers erwartet (Schleiter und Da Silva Zech 2020, S. 3). Daher stiegen die Anforderungen in diesem Bereich vor allem in den Berufsgruppen mit zuvor vergleichsweise geringen Qualifikationsanforderungen an (ebd.). Kompetenzen im Kontext digitaler Technologien besitzen aber nicht nur hinsichtlich der Teilhabe am Arbeitsleben eine hohe Relevanz, sondern stellen auch eine Grundvoraussetzung dar, um am gesellschaftlichen Leben partizipieren zu können (D21 2022). So nehmen bspw. die Anforderungen an die Informationskompetenz im Kontext neuer Technologien zu (Zabal et al. 2014). Wie die PIAAC-Studie (Programme for International Assessment of Adult Competencies) zeigte, verfügen Personen mit einem niedrigen formalen Bildungsniveau über eine geringere technologiebasierte Problemlösungskompetenz (Ziegler 2017). Deshalb ist Weiterbildung für gering Qualifizierte in diesem Bereich besonders bedeutend, um ihrer Exklusion in einer digitalisierten Gesellschaft entgegenzuwirken (Nüßlein und Schmidt 2020).

Obwohl sich durch die Covid-19-Pandemie eine größere Polarisierung der Teilnahme an digitalen Lernangeboten zwischen niedriger und höher qualifizierten Erwachsenen als vor der Pandemie zeigte (Ehlert et al. 2021), wird an das Lernen mittels digitaler Medien häufig die Hoffnung geknüpft, dass sich bestehende Ungleichheiten der Weiterbildungsbeteiligung nivellieren lassen (Bellmann und Leber 2021). Denn in Hinblick auf gering Qualifizierte werden individualisierbare, praxisorientierte und flexible Lernarrangements, beispielsweise mit digitalen Medien, als zielführend beschrieben, um Barrieren wie Lernentwöhnung, fehlende Lernmotivation, beispielsweise aufgrund negativer Lernerfahrung, Versagensängste und Zeitdruck in Präsenzveranstaltungen sowie finanzielle und organisatorische Hürden abzubauen (Pfeiffer 2017; Seyda 2019). Gleichzeitig verweisen empirische Untersuchungen darauf, dass Personen mit niedrigem (42 %) bzw. keinem Schulabschluss (44 %) rund viermal häufiger keine Lernerfahrung mit digitalen Medien besitzen als Erwachsene mit hohem Schulabschluss (10 %) (BMBF 2020, S. 35). Ursache dafür könnte eine höhere Skepsis gering Qualifizierter gegenüber digital gestützten Lernangeboten sein. Zudem wird von dieser Zielgruppe häufig der Mehrwert von (digitalen) Weiterbildungsangeboten unterschätzt (ebd.). Entsprechend dem „Innovativeness-Needs-Paradox“ (Rogers 1983, S. 263) nutzen damit gerade die Personen die (möglichen) Vorteile der Digitalisierung am wenigsten, die sie am meisten brauchen würden:

„Those individuals or other units in a social system who most need the benefits of a new technological idea (the less educated, less wealthy, and the like) are generally the last to adopt that innovation. The units in a system who adopt first generally least need the benefits of the innovation. This paradoxical relationship between innovativeness and the need for benefits of an innovation tends to result in a wider socioeconomic gap between the higher and lower socioeconomic individuals in a social system. Thus, one consequence of many technological innovations is to widen socioeconomic gaps in a social system“ (Rogers 1983, S. 263 f.).

Die vorliegenden Studienergebnisse untermauern damit auch Grundannahmen, wie sie im Zusammenhang mit Theorien „digitaler Ungleichheit“ formuliert wurden. Demnach profitieren sozioökonomisch besser positionierte Gesellschaftsmitglieder mehr von neuen Entwicklungen als diejenigen, die bereits hinsichtlich der materiellen, geistigen, zeitlichen, sozialen und kulturellen Ressourcen benachteiligt sind (van Dijk 2005). So nimmt, wie dargestellt, mit steigendem erreichtem schulischem bzw. beruflichem Abschluss neben der Weiterbildungsquote auch die Quote der Teilnahme an Bildung mit digitalen Medien zu (BMBF 2020).

Eine Behebung dieser Ungleichheit ist nicht nur über die Verfügbarkeit der Technologien zu lösen, sondern erfordert auch die entsprechenden Kompetenzen zur Wahrnehmung und Nutzung damit verbundener Chancen (Zillien 2009). Daraus lässt sich schlussfolgern, dass digitale Weiterbildungsangebote so gestaltet werden müssen, dass gering Qualifizierte Kompetenzen im Bereich digitaler Medien entwickeln, den Mehrwert digitaler Technologien erkennen und Barrieren wie Skepsis gegenüber digitalen Medien sowie Kosten und Risiken der Weiterbildungsteilnahme abgebaut werden können. Dafür sind die Angebote zielgruppenspezifisch zu gestalten (Klös et al. 2020). Diese Anforderungen sind vor dem Hintergrund zu bewerten, dass „in fast jeder dritten formalen Bildungsaktivität und in immerhin jeder fünften Weiterbildungsaktivität Teile des Kursgeschehens ins Internet verlagert [werden] […], um den Teilnehmenden ein raum- und zeitunabhängiges Lernen zu ermöglichen“ (BMBF 2020, S. 55).

Die Verlagerung des Lehr-Lern-Geschehens in den virtuellen Raum sollte daher durch die Unterstützung entsprechender Kompetenzen der Selbststeuerung, die in einem ersten Schritt durch die gering Qualifizierten häufig erst aufgebaut werden müssen, gerahmt werden (Seyda 2019). Darüber hinaus gelten Lernprozessbegleitende, die ein individuelles Feedback sowie eine Beratung sicherstellen, als relevant für die erfolgreiche Weiterbildungsteilnahme gering Qualifizierter (ebd.). Da die Entscheidung für eine Weiterbildungsteilnahme dadurch gekennzeichnet ist, dass deren Folgen nicht unmittelbar, sondern langfristig und insgesamt nur schwer abschätzbar eintreten (Osiander 2017), ist zudem eine individuelle Beratung für die Vermittlung in passgenaue Angebote hilfreich – insbesondere vor dem Hintergrund der Vielzahl an schwer vergleichbaren Weiterbildungsangeboten, der Heterogenität der Zielgruppe und der (informell erworbenen) Kompetenzen sowie struktureller Weiterbildungsbarrieren (Hecker et al. 2017).

Eine Auswertung der vorliegenden Forschungsbefunde zeigte, dass keine Programm- bzw. Angebotsanalysen vorliegen, die explizit auf die Zielgruppe der gering Qualifizierten rekurrieren, um Bedarf und Angebot für diese Zielgruppe passgenau aufeinander abzustimmen. Die Programm- und Angebotsforschung im Bereich der Grundbildung und Alphabetisierungsarbeit (u. a. Mania und Thöne-Geyer 2018) erfasst zwar auch die Gruppe der gering Qualifizierten, deckt aber nur einen Teil der adressierten Zielgruppe ab, da neben gering Literalisierten auch Personen ohne formalen Berufsabschluss oder mit einem nicht mehr nachgefragten bzw. nicht anerkannten Berufsabschluss der Gruppe der gering Qualifizierten angehören (Lacher et al. 2022, S. 11). An dieses Desiderat schließt diese Forschungsarbeit an.

## Methodisches Vorgehen

In der Programmforschung werden mittels Programmanalysen Themenstrukturen und -schwerpunkte sowie deren Ausdifferenzierungen untersucht (Fleige et al. 2018, S. 77). Die auszuwertenden Programme lassen sich häufig auf der Mesoebene ansiedeln, wodurch die Methode die entsprechenden „umgesetzten thematischen Schwerpunkte unter den spezifischen Lernkulturen der Organisation“ (Gieseke 2018, S. 23) in die Analyse einbezieht. Als Unterkategorie von Programmen sowie auf der Mikroebene verortet gelten Angebote, die die konkrete Lehr-Lern-Situation und ihre Gestaltung umfassen (ebd.). Daher „benennt und beschreibt [ein Angebot] mit entsprechenden Ankündigungen einen Inhalt und eine Kompetenz und spricht gegebenenfalls Zielgruppen an“ (ebd., S. 19). Ein Angebot verfolgt das Ziel, spezielle Bedarfe und Bedürfnisse zu bedienen (ebd.). Da sich die Forschungsfragen dieser Arbeit auf mikrodidaktische Aspekte beziehen, bietet sich eine Angebotsanalyse zur Untersuchung an.

Für die Erhebung der Daten wird eine Weiterbildungsdatenbank genutzt. Dieses Vorgehen liegt darin begründet, dass immer mehr Weiterbildungsanbieter ihre Programme auch oder nur noch online zur Verfügung stellen (Käpplinger 2011). So vermarkten nach der Anbieterbefragung wbmonitor (Christ et al. 2020) 97 % ihr Programm über eine eigene Internetpräsenz. 80 % haben oder planen eine Buchung ihrer Kurse über die eigene Website und fast ebenso viele stellen ihre Angebote in eine Weiterbildungsdatenbank ein (ebd., S. 35). Die Covid-19-Pandemie dürfte auch diese Entwicklung weiterführend befördert haben, da aufgrund sich ständig ändernder gesetzlicher Beschränkungen sowie damit notwendiger kurzfristiger Anpassungen der Angebote eine schnellere Editierbarkeit von diesen notwendig wurde.

Trotz bekannter Potenziale der Nutzung von Weiterbildungsdatenbanken im Rahmen von Angebotsanalysen (Kaluza und Brandt 2016) sind keine Forschungsvorhaben bekannt, in der Weiterbildungsdatenbanken als Datengrundlage genutzt wurden. Für die hier zu klärenden Forschungsfragen bieten sie den Vorteil, dass sie im Gegensatz zu bestehenden Programmarchiven keine regionale Begrenzung aufweisen (z. B. Programmarchiv der Humboldt-Universität Berlin für die Bundesländer Berlin und Brandenburg^3^) oder auf einzelne Anbieter beschränkt sind (z. B. Programmarchiv des Deutschen Instituts für Erwachsenenbildung auf Volkshochschulen^4^).

Da in dieser Forschungsarbeit die Zielgruppe der gering Qualifizierten adressiert wird, wurde die Datenerhebung mithilfe der Weiterbildungsdatenbank KURSNET der Bundesagentur für Arbeit (BA) realisiert. KURSNET wurde im Zuge der Nationalen Weiterbildungsstrategie in das Portal der BA^5^ integriert und besitzt als „Deutschlands größte Onlineplattform für Weiterbildungen“ (BMAS und BMBF 2019, S. 7) eine besondere Relevanz. Die Weiterbildungsdatenbank bietet eine Übersicht von „über 4,5 Mio. Bildungsangeboten“ (ebd.) bei akkreditieren Weiterbildungsträgern und kann kostenfrei zur Angebotsveröffentlichung und -suche genutzt werden (BMAS und BMBF 2019). Dadurch besteht eine tagesaktuelle und schnelle Übersicht über lokale, regionale und überregionale Angebote. Durch die Eingabe eines Schlagworts und die Nutzung unterschiedlicher Filter können die Suchergebnisse zielgerichtet eingeschränkt werden. Hierbei werden die Programmbereiche in KURSNET durch eine Systematik^6^ abgebildet, durch die die Bildungsangebote in der Datenbank zugeordnet werden (BA 2022a, S. 18). Diese Systematik „ist ein Ordnungssystem aus hierarchisch gegliederten Systematikpositionen, dass [sic] es ermöglicht, innerhalb von KURSNET inhaltlich vergleichbare Bildungsangebote thematisch in Gruppen zusammenzufassen“ (ebd., S. 66). Eine standardisierte Darstellung der Angebote unterstützt den Vergleich zwischen den Angeboten (Bellmann und Dummert 2016). Unter den Weiterbildungsanbietern hat KURSNET eine herausgehobene Stellung inne: 82 % der Einrichtungen, die mithilfe von Datenbanken Weiterbildungsangebote veröffentlichen, nutzen dafür (auch) KURSNET (Christ et al. 2020, S. 38). Unter den Einrichtungen mit einem Schwerpunkt im Bereich der nach dem Sozialgesetzbuch (SGB) geförderten Arbeitsmarktdienstleistungen sind es sogar 99 % (ebd.). Daher bietet sich KURSNET zur Datenerhebung für die Zielsetzung dieser Studie an.

**Figure 1:**
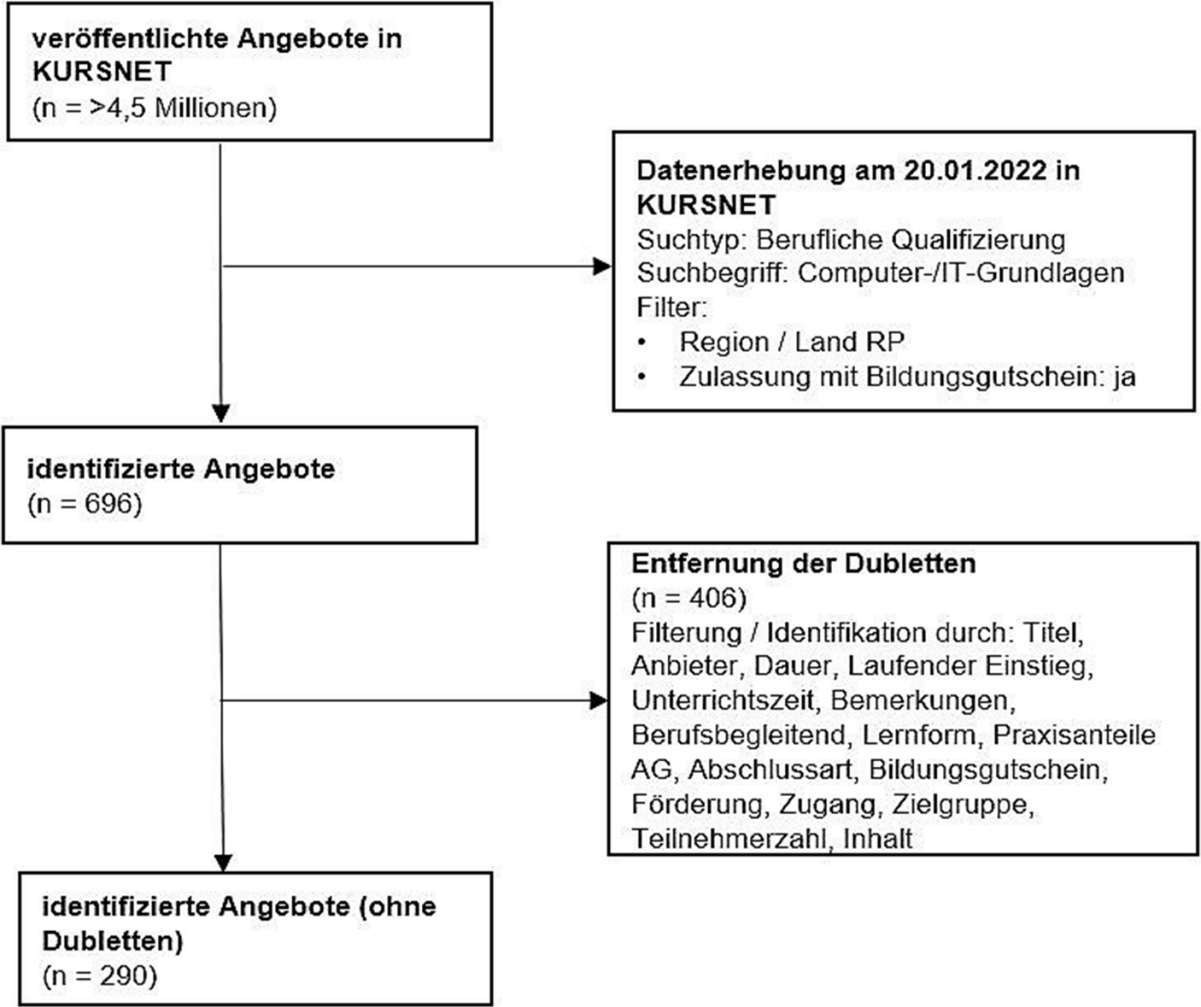

Zur Erfassung relevanter Angebote wurde in KURSNET eine automatisierte Datenerhebung mithilfe des Web Scrapers Selenium^7^ und dem WebDriver ChromeDriver^8^ durchgeführt^9^. Auf Basis der festgelegten Suchbegriffe und Filter wurden die zugehörigen URLs der jeweiligen Suchergebnisse automatisiert geöffnet und die für die Analyse relevanten Informationen der Angebote aus den entsprechenden Web-Elementen extrahiert. Anschließend wurden die Daten in das Tabellenkalkulationsprogramm Microsoft® Excel als CSV-Datei für die Datenauswertung importiert. Abb. 1 visualisiert den Prozess der Datenerhebung sowie die Selektion der analysierten Angebote.

Zur Identifikation relevanter Angebote in KURSNET wurde als ein Filter der Suchtyp „Berufliche Qualifizierung“ gewählt. Die entsprechenden Kurse sind im Gegensatz zu dem optional wählbaren Suchtyp „Aufstiegsweiterbildung“ nicht gesetzlich geregelt und dienen „zum Erwerb bzw. zur Auffrischung, Ergänzung oder Erweiterung von bereits vorhandenen beruflichen Kenntnissen und Qualifikationen“ (BA 2022a, S. 151). Zur Eingrenzung von Angeboten für die ausgewählte Zielgruppe wurden nur solche ausgewählt, die mit einem Bildungsgutschein gefördert werden. Dieser wird durch die BA bzw. das Jobcenter an Arbeitslose, Arbeitsuchende oder Beschäftigte ausgegeben, ermöglicht die Übernahme der Weiterbildungskosten Förderberechtigter und verfolgt das Ziel, Arbeitslosigkeit zu beenden, eine drohende Arbeitslosigkeit abzuwenden bzw. das Nachholen eines Schulabschlusses zu ermöglichen (BA o.J.). Darüber hinaus werden bei arbeitslosen Arbeitssuchenden auch Maßnahmen gefördert, die „zu einer Kompetenzerweiterung und Verbesserung der Beschäftigungsmöglichkeiten führen“ (ebd.). Des Weiteren fokussiert der Bildungsgutschein auf den Erwerb von Grundkompetenzen, u. a. im Bereich der Informations- und Kommunikationstechnologien, stets mit Orientierung an dem Bedarf des Arbeitsmarktes (ebd.). Damit werden mit dem Bildungsgutschein nach der vorgestellten Definition Personen adressiert, die aufgrund ihrer Qualifikationen nicht oder nicht mehr über die am Arbeitsmarkt nachgefragten Qualifikationen verfügen. Zudem wurde aufgrund des Datenumfangs die Analyse auf das Bundesland Rheinland-Pfalz (RLP)^10^ beschränkt. Inhaltlich wurde die Analyse auf Angebotsankündigungen aus dem Themenfeld digitale Kompetenzen fokussiert, welche aus den erwähnten Gründen von übergreifender und aktueller Bedeutung für die Zielgruppe sind. So gelten bspw. fehlende Kompetenzen im Bereich der Office-Programme als „ein Hindernis für berufliche Weiterentwicklung sowie Teilhabe“ (D21 2021, S. 55).

**Figure 2:**
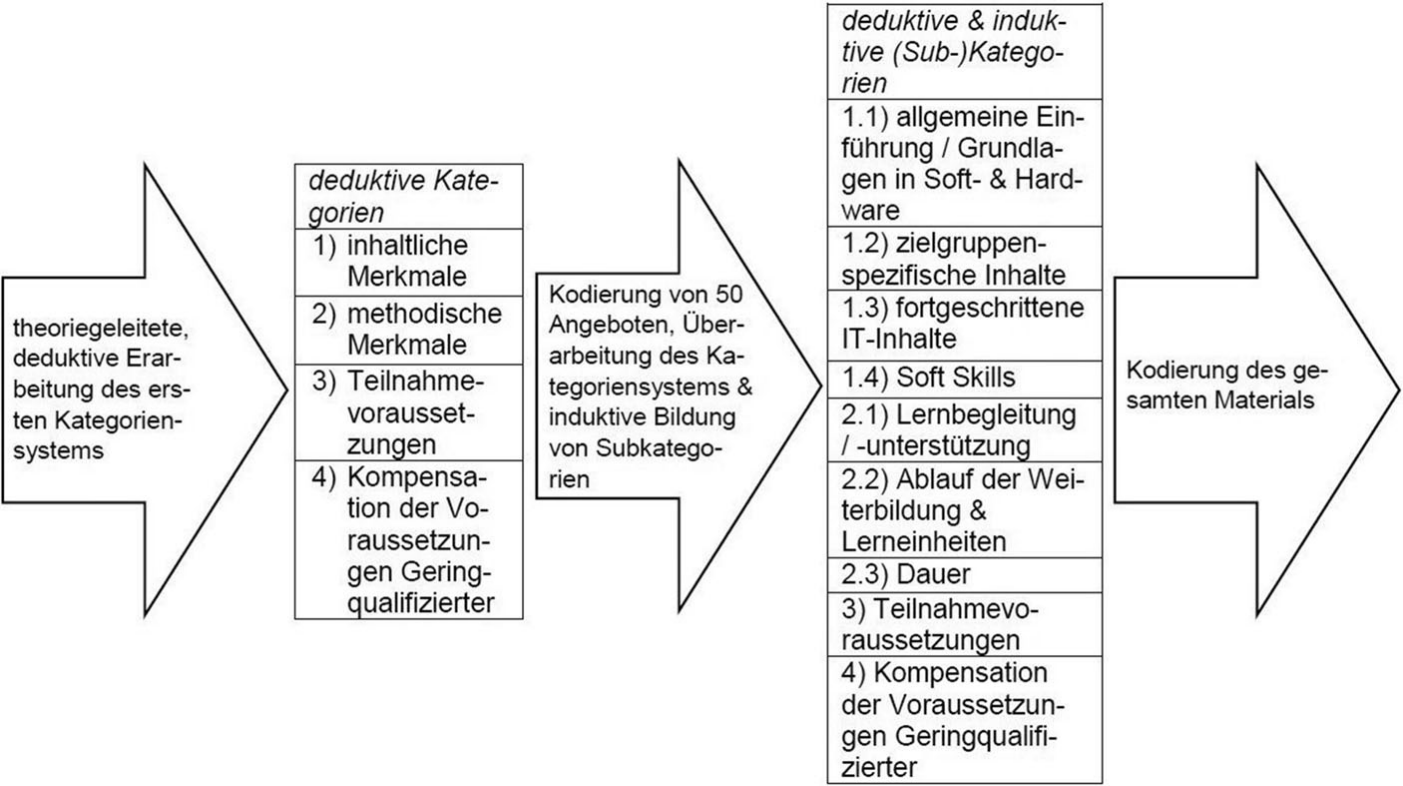

Mithilfe des Suchbegriffs „Computer‑/IT-Grundlagen“ (aus der KURSNET-Systematik der Schlagworte) konnten 696 Weiterbildungsangebote identifiziert werden. Diese wurden automatisch aus der Datenbank heruntergeladen und gespeichert (vgl. Abb. 1). Nach der Datenerhebung und dem Import in Microsoft® Excel wurden die Suchergebnisse anhand festgelegter Kriterien um Dubletten bereinigt, wodurch 290 relevante Angebote identifiziert werden konnten. Anschließend wurden die Angebote mithilfe der strukturierenden Inhaltsanalyse nach Mayring (1995, S. 78) und der Software MAXQDA quantitativ und qualitativ ausgewertet, wobei das Ziel einer inhaltlichen Strukturierung (ebd., S. 79) verfolgt wurde. Hinsichtlich der Auswertung bietet Abb. 2 eine vereinfachte Übersicht über die Erarbeitung der Kategoriensysteme und deren Inhalte.

## Ergebnisse

Die analysierten Angebote stammen von 21 Weiterbildungsanbietern, die alle privat-kommerziell tätig sind. Im Rahmen der Analyse zeigt sich, dass ca. die Hälfte der Angebote von nur zwei Anbietern bereitgestellt werden. Dennoch ist insgesamt eine ausreichende Heterogenität gegeben, die die Breite der Weiterbildungsanbieter in diesem Segment widerspiegelt.

Bezüglich der ersten Forschungsfrage *(Welche inhaltlichen und didaktischen Merkmale weisen Weiterbildungsangebote mit Bezug zur Digitalisierung für gering Qualifizierte auf?)* bietet sich durch die Analyse eine Einteilung der Angebote in drei inhaltliche Kategorien an:

1. Angebote mit dem Ziel des Erwerbs von digitalen Grundkompetenzen für eine allgemeine Zielgruppe (63,6 % der Angebote),
2. Angebote mit dem Ziel des Erwerbs von digitalen Grundkompetenzen mit Berufsbezug (34,3 %) und
3. Angebote, die fortgeschrittene IT-Kompetenzen mit Berufsbezug fokussieren (2,1 %).

Eine Gemeinsamkeit aller Angebote ist, dass ausschließlich das Betriebssystem von Microsoft® Windows sowie die Microsoft® Office-Programme thematisiert werden.

In den Angebotsbeschreibungen der ersten Kategorie „Ziel des Erwerbs von digitalen Grundkompetenzen für eine allgemeine Zielgruppe“ (144 Kurse) steht die Anwendung der PC-Hardware sowie ausgewählter Microsoft® Office-Software im Fokus. In den Angebotsbeschreibungen werden die Digitalisierung, die digitale Transformation sowie grundlegende digitale Kompetenzen als notwendig und zielführend für die Bewältigung des (beruflichen) Alltags betont.

In den Kursbeschreibungen der zweiten Kategorie „Ziel des Erwerbs von digitalen Grundkompetenzen mit Berufsbezug“ (99 Kurse) wird die digitale Transformation dagegen als Chance für einen beruflichen Neuanfang sowie eine Höherqualifizierung thematisiert. Tab. 1 bietet einen Überblick über die Kategorie und deren Subkategorien.

**Table 1:** 

Subkategorien der Angebote mit ggf. weiterer Unterteilung		Kursinhalte	Adressatinnen und Adressaten	Teilnahmevoraussetzungen	Formulierte Ziele gemäß Angebotsbeschreibungen
Kurse mit der Zielgruppe von Personen *ohne Berufsausbildung* (80 Kurse)	Kurse für *allgemeine berufliche Anwendungen* (19 Kurse)	Anwendung der PC-Hardware sowie ausgewählter Microsoft® Office-Software	Personen ohne Berufsausbildung	Keine PC-Vorkenntnisse erforderlich	Erwerb von Grundlagen für die berufliche Anwendung sowie Verwertung dieser neu erworbenen Kenntnisse
				Grundlegende Deutsch-Sprachkenntnisse (A2 bzw. B1/B2)	
	Kurse mit einem *Bezug zum Ausbildungsbereich IT/kaufmännisch/Büro* (34 Kurse)	Anwendung der PC-Hardware sowie ausgewählter Microsoft® Office-Software	Personen ohne Berufsausbildung	Keine bzw. grundlegende PC-Kenntnisse erforderlich	Erwerb von Grundlagen für die berufliche Anwendung sowie Verwertung dieser neu erworbenen Kenntnisse
				Grundlegende Deutsch-Sprachkenntnisse (A2 bzw. B1/B2)	
	Kurse mit einem Bezug zu *sozialen und medizinischen Berufen* (27 Kurse)	Anwendung der PC-Hardware sowie ausgewählter Microsoft® Office-Software	Arbeitssuchende und Berufsrückkehrende (Berufsausbildung keine Voraussetzung)	Keine bzw. wenige Kenntnisse im Umgang mit dem PC erforderlich	Kompensation fehlender spezifischer arbeitsmarktrelevanter Kompetenzen im IT-Bereich
				Interesse an medizinischen oder sozialen Themen	
				Grundlegende Deutsch-Sprachkenntnisse (mindestens A2)	
Zielgruppe von Personen *mit Berufsausbildung bzw. -erfahrung *(19 Kurse)		u. a. Datenschutz, Verschlüsselung, Programme und Algorithmen sowie Virenschutz	Personen mit Berufsausbildung bzw. -erfahrung	Berufsausbildung bzw. -erfahrung	Kompensation fehlender berufsspezifischer Kompetenzen der Digitalisierung
				Grundlegende PC-Kenntnisse	
				Grundlegende Deutsch-Sprachkenntnisse	
				Kenntnisse der Microsoft® Office-Software	
		Kurse mit einem klaren Berufsbezug zu dem Ausbildungsbereich IT/kaufmännisch/Büro	Personen, die bereits am Arbeitsmarkt partizipiert bzw. eine Ausbildung oder ein (abgebrochenes) Hochschulstudium absolviert haben	Berufsausbildung bzw. -erfahrung	Kompensation fehlender berufsspezifischer Kompetenzen der Digitalisierung
				Gute PC-Kenntnisse	
				Gute Deutsch-Sprachkenntnisse	
				Teilweise grundlegende Englischkenntnisse	

Die identifizierten Kurse der dritten Kategorie, die dem Erwerb von fortgeschrittenen IT-Kompetenzen mit Berufsbezug (6 Kurse) zugeordnet wurden, bieten sowohl Angebote für Personen ohne Berufsausbildung (2 Kurse) als auch Kurse, deren Teilnahme eine Ausbildung oder ein (abgebrochenes) Hochschulstudium erfordert (4 Kurse). Die im Vergleich zu den anderen Kursen komplexen Inhalte werden mit einem klaren Berufsbezug vermittelt. Grundsätzlich wird in den Angebotsbeschreibungen aller Kategorien deutlich, dass eine kritische Auseinandersetzung mit der Nutzung digitaler Medien und die Folgen der Digitalisierung nur marginal adressiert werden.

Hinsichtlich der Formate der Angebote zeigte sich, dass 78,3 % in Voll- und 21,7 % in Teilzeit angeboten werden. Während die Kurse der Kategorie „Erwerb von fortgeschrittenen IT-Kompetenzen mit Berufsbezug“ für Erwerbslose mit IT-Grundkompetenzen lediglich in Vollzeit angeboten werden, sind Kurse für den Erwerb von digitalen Grundkompetenzen auch in Teilzeit verfügbar. Es fällt auf, dass die Angebote mit steigendem Kompetenzniveau nicht an Flexibilität zunehmen, um bspw. eine bessere Vereinbarkeit mit Beruf und Familie zu fördern, da von einer gesteigerten Selbstlernkompetenz ausgegangen werden kann. Stattdessen ist die didaktische Gestaltung hinsichtlich Regelung der Lehr-Lern-Zeiten, Anwesenheit sowie Wissensvermittlung konsistent.

**Figure 3:**
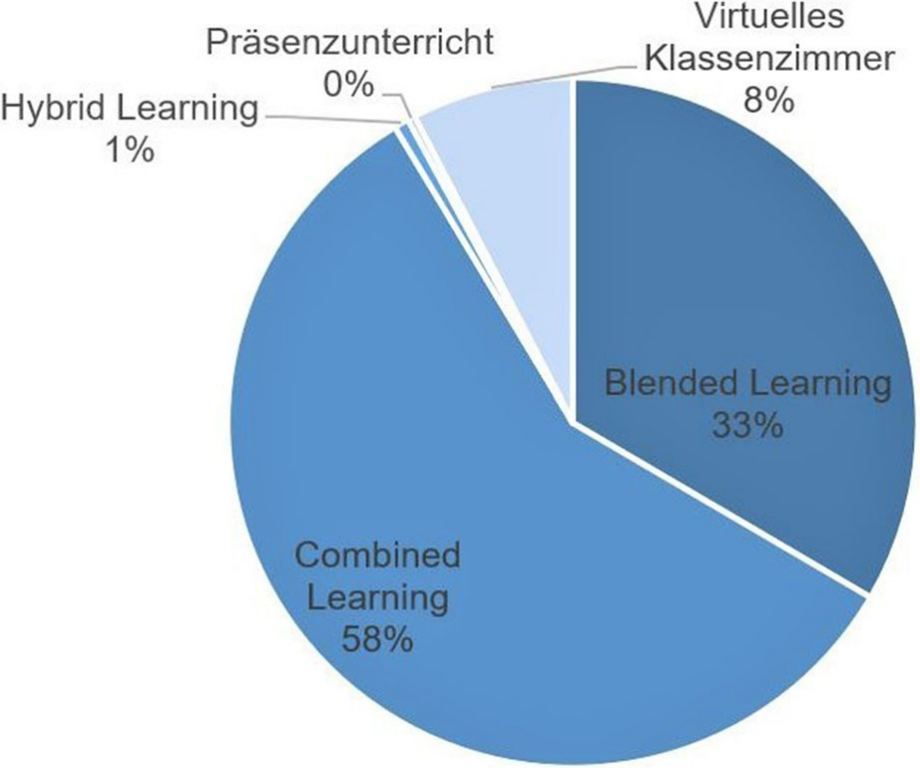

Bezüglich der Analyse der Lernformen visualisiert Abb. 3 eine unterschiedliche Verteilung jener:

Auffällig ist, dass kein reiner Präsenzunterricht vor Ort angeboten wird. Alle Anbieter stellen aber eine digitale und interaktive Lernumgebung in Form einer Online-Akademie zur Verfügung. In der Online-Akademie besteht bei allen angebotenen Lernformen sowohl für den Präsenzunterricht als auch für die individuelle, d. h. ohne angeleiteten Unterricht stattfindende Wissensvertiefung ein festgelegter Stundenplan sowie Anwesenheitspflicht in der Lernumgebung. Dabei stellen die Combined-Learning-Angebote interaktive Lehrveranstaltungen dar, bei denen sich Teilnehmende sowohl vor Ort als auch online mittels einer Videokonferenzsoftware beteiligen und miteinander interagieren können (BA 2022b, S. 61). Blended-Learning-Angebote beinhalten einen Mix aus E‑Learning-Angeboten und Präsenzveranstaltungen, während Hybrid Learning die beiden vorhandenen Lernformen des Blended und Combined Learning miteinander verbindet (ebd.). Im Rahmen der Analyse fällt v. a. auf, dass Blended-Learning-Angebote im Gegensatz zu Angeboten der anderen beiden Lernformen durch eine zusätzliche Online-Lernplattform mit Fachmaterial ergänzt werden.

Die Teilnehmenden können bei allen Angeboten jeweils auswählen, ob sie von zuhause oder von dem jeweiligen Bildungsstandort aus an der Weiterbildung teilnehmen möchten. Die Kurse folgen einem modularen Aufbau und sind somit je nach Vorkenntnissen der Adressatinnen und Adressaten individuell kombinierbar. Bei den Kursen, welche den Kategorien „Erwerb von digitalen Grundkompetenzen für eine allgemeine Zielgruppe“ sowie dem „Erwerb von digitalen Grundkompetenzen mit Berufsbezug“ zugeordnet wurden, wird in den Angebotsbeschreibungen die Praxisnähe durch das Trainieren von Arbeitssituationen betont. Zudem wird als Teil der Konzepte auch das Erlernen sowie Trainieren der Selbstlernkompetenz mit digitalen Medien genannt.

Betrachtet man bezugnehmend auf die zweite Forschungsfrage *(Was wird für eine Teilnahme vorausgesetzt (z.* *B. technische Ausstattung, medienbezogene Kompetenzen)?)* die Voraussetzungen für die Teilnahme an den Weiterbildungsangeboten, so wird deutlich, dass die Anforderungen hinsichtlich der Medienkompetenz und dem deutschen Sprachniveau, weiteren grundlegenden Sprachkenntnissen in Englisch sowie dem Ausbildungsgrad mit Zunahme der inhaltlichen Komplexität und mit verstärktem Berufsbezug ansteigen. Die Teilnehmenden können bei allen Anbietern technisches Equipment vor Ort nutzen. Wenn die Teilnehmenden von zuhause aus an den Weiterbildungsangeboten partizipieren möchten, wird nur bei einem Teil der Kurse die Homeoffice-Ausstattung gestellt. Dies sowie der Modus des Beratungsgesprächs bzw. Auswahlverfahrens unterscheidet sich je nach Kurskategorie. Tab. 2 bietet hierzu eine Ergebnisübersicht.

**Table 2:** 

Kurskategorie	Anteil der Kurse, in denen eine Homeoffice-Ausstattung gestellt wird (%)	Modus des Beratungsgesprächs/Auswahlverfahrens im Vorfeld der Maßnahme
*Vermittlung von digitalen Grundkompetenzen*	56,8	Beratungsgespräch verpflichtend; kein Auswahlverfahren
*Vermittlung von digitalen Grundkompetenzen mit Berufsbezug*	27,3	Beratungsgespräch optional; kein Auswahlverfahren
*Fortgeschrittene IT-Kompetenzen*	0	Kein Beratungsgespräch; verpflichtendes internes Auswahlverfahren

Diese Befunde sind auch hinsichtlich der dritten Forschungsfragestellung *(Wie werden die fehlenden oder unzureichenden Voraussetzungen der Zielgruppe für die Weiterbildungsangebote kompensiert?) *von Bedeutung: Die fehlenden oder unzureichenden Voraussetzungen der Zielgruppe werden für die Weiterbildungsangebote hinsichtlich der Ausrüstung für ein Lernen von zuhause aus und der Medienkompetenzen vor allem bei jenen Angeboten kompensiert, in denen IT-Grundlagen und digitale Grundkompetenzen vermittelt werden. Wie bereits dargestellt, besteht vor Ort aber bei allen Anbietern in den jeweiligen Einrichtungen und Zweigstellen die Möglichkeit, technisches Equipment zu nutzen. Zudem werden Vorkenntnisse, informell erworbene Kompetenzen sowie die Ziele der Personen durch persönliche Beratungsgespräche bei den Weiterbildungsanbietern erfasst. Darüber hinaus wurden folgende Gestaltungsaspekte der untersuchten Weiterbildungsangebote identifiziert, welche förderlich für das Adressieren sowie die anschließende Weiterbildungsteilnahme der gering Qualifizierten sein könnten, da sie sowohl auf potenzielle Defizite der Zielgruppe fokussieren (z. B. unzureichende Vorkenntnisse, fehlende Ausstattung) als auch auf deren grundsätzliche Heterogenität:

- die interaktive Gestaltung der Angebote,
- die Nennung von Ansprechpartnern (z. B. Dozierende, Lernbegleitende), die jederzeit über die Online-Plattform erreichbar sind,
- das Erlernen sowie die Förderung von Selbstlernkompetenz mit digitalen Medien während der Weiterbildung,
- ein modularer Aufbau der Weiterbildungsangebote, welcher eine Wahl nach individuellen Interessen sowie das schnelle Erwerben eines arbeitsmarktrelevanten Zertifikats ermöglicht,
- die Betonung eines Verwendungszusammenhangs bzw. des Mehrwerts der Weiterbildung sowie der zeitgemäßen Inhalte und verbesserter Jobchancen,
- die Praxisnähe der Inhalte,
- der feste Stundenplan für ein vereinfachtes Zeitmanagement,
- die interaktive Gestaltung der Weiterbildung durch wechselnde Unterrichtsinhalte,
- die Einführung in die Online-Lernumgebung der einzelnen Weiterbildungsangebote sowie
- die Bereitstellung der technischen Ausstattung bei den Weiterbildungsanbietern vor Ort sowie teilweise für die Nutzung zuhause.

Durch die Förderung mit einem Bildungsgutschein wird die Finanzierung der Weiterbildung in allen Angebotsbereichen sichergestellt.

## Diskussion

Für die Förderung mit einem Bildungsgutschein müssen sowohl die Träger als auch deren Maßnahmen ein kriterienbasiertes Akkreditierungsverfahren^11^ nach einem festgelegten Schema durchlaufen. Obwohl auch öffentlich geförderte Weiterbildungsanbieter wie Volkshochschulen (VHS) in RLP solche Akkreditierungsverfahren durchlaufen und entsprechende Weiterbildungsmaßnahmen bereitstellen^12^, deuten die identifizierten Angebote der ausschließlich privat-kommerziell tätigen Anbieter darauf hin, dass öffentliche Träger die Angebote des betrachteten Themenbereichs nicht in KURSNET einpflegen. Trotz der Vielzahl der Anbieter, die bei der Analyse berücksichtigt wurden, weisen die Angebote ähnliche Titel und Schwerpunkte auf. Eine Begründung hierfür könnte das bereits dargestellte Akkreditierungsverfahren der Maßnahmen und Träger als Voraussetzung für die Förderungsmöglichkeit mit einem Bildungsgutschein sein. Durch die ähnlichen Titel und Schwerpunkte ist auf der einen Seite eine klare Orientierung für die Teilnehmenden gegeben, auf der anderen Seite wird damit auch die Breite der Angebote und das Adressieren spezifischer Zielgruppen und Berufsfelder eingeschränkt.

Auffällig sind die 27 Kurse für Personen ohne Berufsausbildung mit dem Schwerpunkt soziale und medizinische Berufe. Diese Schwerpunktsetzung könnte darin begründet liegen, dass sich im sozialen Bereich „quantitativ ein steigender Bedarf an personenbezogenen Dienstleistungen […] beobachten [lässt]“ (Thiessen und Borrmann 2018, S. 64–65). Zudem besteht ein „erheblicher Fachkräftemangel“ (Stuckatz und Badel 2015, S. 117) im Pflegebereich, wodurch aktuell und – aufgrund des demografischen Wandels – auch zukünftig ein Fachkräftebedarf besteht, der „durch Absolvierende der dreijährigen Berufsfachschulen nicht kompensiert werden kann“ (ebd.). Aufgrund von Digitalisierungspotenzialen sowie bestehenden digitalen Technologien, die zur Dokumentation, Informationsverarbeitung, Organisation und Kommunikation eingesetzt werden (Fachinger und Mähs 2019), sind sowohl für Berufsrückkehrende als auch Arbeitssuchende Kompetenzen zur Nutzung dieser Technologien notwendig.

Insgesamt wird deutlich, dass durch die Kurse im Bereich Computer‑/IT-Grundlagen sowohl Personen angesprochen werden, die konkrete Berufe im Bereich der Informationstechnik anstreben als auch Personen, die Kompetenzen im Bereich Computer/IT grundsätzlich für ihren Wiedereinstieg in den Arbeitsmarkt sowie ihren Alltag benötigen. Dies ist vor allem vor dem Hintergrund der dargestellten Heterogenität der Zielgruppe positiv zu bewerten. Denn auf diese Weise können passende Kurse, orientiert an den persönlichen Zielen und individuellen Voraussetzungen, ausgewählt werden und die Teilhabe gering Qualifizierter am digitalisierten Arbeitsmarkt gefördert werden.

Festzustellen ist weiterhin eine deutliche Verbindung von Qualifizierungszielen und unterschiedlichen formalen Bildungsniveaus: Während in Kursen zum Erwerb von digitalen Grundkompetenzen für eine allgemeine Zielgruppe mit und ohne Berufsbezug Personen ohne Berufsausbildung der grundlegende Umgang mit der Hard- und Software vermittelt werden soll, werden in den Kursen mit Berufsbezug für Personen mit Berufsausbildung bzw. -erfahrung durch die Kursinhalte Datenschutz, Verschlüsselung und Virenschutz Aspekte thematisiert, die zu einer gesteigerten digitalen Souveränität beitragen sollen. Diese Selektion anhand des Bildungsniveaus ist insofern kritisch zu betrachten, da hierdurch einerseits solche Personen von fortgeschrittenen Kursen ausgeschlossen werden, die eine Berufsausbildung absolviert haben, die nicht (mehr) anerkannt wird, oder die über Berufserfahrung verfügen, für die entsprechende Nachweise fehlen (z. B. durch eine Flucht). Somit wäre zu überdenken, die Verfahren stärker an den tatsächlichen Kompetenzen auszurichten und hier z. B. entsprechende Tests einzusetzen.

Positiv hervorzuheben sind in diesem Zusammenhang die teilweise verpflichtenden Beratungsgespräche, durch die der individuelle Nutzen der Weiterbildungsteilnahme und die Vermittlung in passgenaue Angebote gefördert wird. Es ist daher zu erwarten, dass durch ein verpflichtendes Beratungsgespräch für alle Teilnehmenden arbeitsmarktbezogene Defizite besser reflektiert und passgenauere Angebote ausgewählt werden können.

Hinsichtlich der didaktischen Merkmale zeigt sich v. a. bei den Kursen der Kategorie „Ziel des Erwerbs von digitalen Grundkompetenzen für eine allgemeine Zielgruppe“, dass u. a. überfachliche und persönliche Kompetenzen wie die Arbeit im Team trainiert werden, die am Arbeitsmarkt ebenfalls bedeutend sind (Stahl-Rolf et al. 2018, S. 15). So zeigte eine Analyse von 1011 Online-Stellenanzeigen, dass „Zuverlässigkeit, Flexibilität und Teamfähigkeit […] auch in digitalisierten Zeiten ausschlaggebend für eine erfolgreiche Jobsuche [sind]“ (Schöpper-Grabe und Valhaus 2017). An die besonderen Bedarfe der Zielgruppe anschließend bildet in dieser Kategorie auch das Erlernen sowie Trainieren der Selbstlernkompetenz mit digitalen Medien einen Teil des Konzepts, trainiert also weitere überfachliche Kompetenzen und begegnet so der teilweise nur mangelhaft vorhandenen Erfahrung im Umgang mit digitalen Medien in dieser Zielgruppe. Daher kann dieses Kurskonzept die Partizipation von gering Qualifizierten in der digitalisierten Arbeitswelt sowie Gesellschaft fördern. Gleichzeitig sollte stets transparent sein, wann und auf welche Weise Dozierende erreichbar sind, damit bei Bedarf diese Unterstützungsmöglichkeit auch genutzt werden kann. Kritisch betrachtet werden kann die Voraussetzung eines steigenden deutschen Sprachniveaus mit Zunahme der Komplexität der Inhalte. So werden Personen, deren Abschluss in Deutschland nicht anerkannt wird, von den anspruchsvolleren Weiterbildungen ausgeschlossen, obwohl diese Personen durchaus über ausreichendes Wissen und ausreichende Kompetenzen verfügen können. An dieser Stelle wird erneut deutlich, dass die Diversität der Zielgruppe nur zum Teil bei der Angebotsgestaltung Berücksichtigung findet, wodurch eine individuelle Förderung und bestmögliche Integration in einen Arbeitsmarkt mit steigenden Anforderungen im Kontext der Digitalisierung behindert werden. Zudem wäre hinsichtlich des Formats der Angebote eine Zunahme der Flexibilität mit steigendem Kompetenzniveau anzustreben, um einerseits die eigenständige Anwendung der erworbenen Kompetenzen (z. B. Selbstlernkompetenz, Umgang mit digitalen Medien, Strukturierung der Lernzeiten, Arbeit im Team) zu festigen und den Personen eine Partizipation am Arbeitsmarkt zu ermöglichen. Personen, die bereits am Arbeitsmarkt partizipieren, haben durch den Großteil der Angebote in Vollzeit sowie das fehlende berufsbegleitende Angebot wenig Möglichkeiten, sich durch diese Weiterbildungen höher zu qualifizieren bzw. sich zu orientieren. Das bedeutet zugleich, dass die Angebote vor allem auf Personen ausgerichtet sind, die keiner Arbeit nachgehen, obwohl durch Bildungsgutscheine geförderte Angebote grundsätzlich auch Beschäftigten offenstehen sollten. Lediglich Kurse für den Erwerb von digitalen Grundkompetenzen sind auch in Teilzeit verfügbar. Dadurch werden auch Berufstätige und Menschen mit familiären Verpflichtungen angesprochen. An dieser Stelle wirken die in dieser Analyse untersuchten Angebote der Exklusion aus einer digitalisierten Gesellschaft nicht klar entgegen. Inhaltlich sind im Gegensatz dazu bei den Kursen, die digitale Grundkompetenzen vermitteln, durchaus Aspekte sichtbar, bei denen die digitalisierungsbezogenen Inhalte für die Weiterbildung und die Steigerung der Partizipation gering Qualifizierter in der digitalisierten Gesellschaft genutzt werden. So werden v. a. grundlegende Kompetenzen vermittelt, die durch den Alltags- bzw. Berufsbezug einen direkten Verwertungszusammenhang aufweisen und die Weiterbildungsbeteiligung fördern können.

Die Ausstattung für ein Lernen von zuhause aus wird nur bei den Kursen zur Verfügung gestellt, die digitale Grundkompetenzen für eine allgemeine Zielgruppe vermitteln. Dass die Ausstattung bei Kursen mit Berufsbezug weniger häufig und bei Angeboten mit fortgeschrittenen IT-Kompetenzen gar nicht gestellt wird, führt zu einer Ungleichheit bei der Möglichkeit der Angebotsteilnahme. Dem kann nur teilweise durch die Bereitstellung der technischen Ausstattung vor Ort in Einrichtungen bei den Weiterbildungsanbietern entgegengewirkt werden, da Teilnehmende z. B. aus ländlichen Regionen mit einer schlechten Infrastruktur des öffentlichen Personennahverkehrs auf diese Weise benachteiligt werden können.

Überraschend ist, dass keine Angebote im Präsenzformat vor Ort identifiziert wurden. Dies könnte mit den Hygiene‑, Test- und Abstandsmaßnahmen aufgrund der Covid-19-Pandemie zusammenhängen. Zum Zeitpunkt der Datenerhebung bestand neben der Maskenpflicht sowie der Kontakterfassung eine Testpflicht, d. h. der Nachweis eines tagesaktuellen, negativ ausgefallenen Antigen-Schnelltests^13^. Hier ist in weiterführenden Forschungen zu klären, ob dies im Zusammenhang mit der Covid-19-Pandemie und deren Kontaktbeschränkungen steht oder aber (auch) pädagogisch oder ökonomisch durch die Anbieter begründet wird. Letzteres erscheint vor allem vor dem Hintergrund der ausschließlichen Betrachtung privatwirtschaftlicher Anbieter interessant.

Hinsichtlich des Samples der dargestellten Angebotsanalyse sind bis auf zwei Anbieter alle Träger bundesweit tätig. Durch die bundeseinheitlichen Regelungen zum Bildungsgutschein und der entsprechenden Akkreditierung der Bildungsanbieter ist zu erwarten, dass die Angebote auch in anderen Bundesländern in gleicher oder vergleichbarer Form angeboten werden, denn sowohl Titel und Kurzbeschreibung der Maßnahme als auch deren Zielsetzung sind Teile des Akkreditierungsverfahrens.^14^

Wesentlich erscheint es daher, für eine weitere Fundierung der Befunde in Bezug auf die Zielgruppe der gering Qualifizierten Angebote in anderen Themenfeldern zu analysieren sowie auch die Angebote der öffentlich geförderten Weiterbildungsträger einzubeziehen.

## Fazit

Die vorliegenden Ergebnisse zeigen, dass im Rahmen des Weiterbildungsangebots im Bereich Computer‑/IT-Grundlagen für gering Qualifizierte drei zentrale inhaltliche Kategorien identifiziert werden können. Diese unterscheiden sich nach inhaltlichem Fokus und Zielgruppe. Auffällig ist dabei der große Anteil an Angeboten für den Bereich der Pflege, welcher über den Fachkräftebedarf sowie über notwendige Kompetenzen für digitale Anwendungen erklärbar ist.

Hinsichtlich der didaktischen Gestaltung erscheint es überraschend, dass ausschließlich Angebote gefunden wurden, die mindestens teilweise im Online-Format stattfinden, weshalb die Betrachtung möglicher Folgen der Digitalisierung hier eine besondere Relevanz hat. Zur Reduzierung damit verbundener exkludierender Wirkungen werden eine Reihe von Kompensationsmaßnahmen angeboten. Dabei wird sich – wie auch bei der grundsätzlichen methodischen und inhaltlichen Gestaltung der Angebote – ausschließlich am formalen Bildungsniveau orientiert. In Bezug auf die Heterogenität der Zielgruppe ist damit jedoch eine unzureichende Differenzierung gegeben, welche vorhandene Kompetenzen nicht ausreichend berücksichtigt und so mögliche Potenziale der (Re‑)Integration in den Arbeitsmarkt nicht vollständig nutzt.

Hieran schließt sich die Frage an, wie die Gestaltung der Angebote von den Anbietern begründet wird und welchen Einfluss rechtliche und zertifizierungsrelevante Fragen haben. Als weiteres Forschungsdesiderat wäre zu klären, inwiefern die Kompensationsmaßnahmen wirken und mögliche Zugangsbarrieren zu (Online‑)Weiterbildung verringern. Zudem wäre eine Evaluation am Ende der Weiterbildungsmaßnahmen gewinnbringend, um festzustellen, ob und wie sich die durch die Weiterbildung erworbenen Kompetenzen zur Digitalisierung die beruflichen und gesellschaftlichen Teilhabemöglichkeiten ausgewirkt haben.

Hinsichtlich der Nutzung von Weiterbildungsdatenbanken für die Datenerhebung zeigte sich, dass dieses Vorgehen durchaus Potenziale, aber auch Limitationen bietet. Die Reflexion dessen konnte im Rahmen dieses Beitrags nicht geleistet werden, sollte jedoch aufgrund der steigenden Relevanz von Weiterbildungsdatenbanken Gegenstand zukünftiger Forschung sein.

## Footnotes

1 https://www.arbeitsagentur.de/kursnet.

2 https://www.sowi.uni-kl.de/paedagogik/forschung/projekte-erwachsenenbildung/projektuebersicht/forward.

3 https://www.erziehungswissenschaften.hu-berlin.de/de/ebwb/weiterbildungsprogrammarchiv.

4 https://www.die-bonn.de/weiterbildung/archive/programmplanarchive/default.aspx.

5 https://www.arbeitsagentur.de/.

6 Die Systematik der Schlagworte in KURSNET stellt ein internes Dokument dar und wurde durch die KURSNET-Redaktion zur Verfügung gestellt.

7 https://www.selenium.dev/.

8 https://chromedriver.chromium.org/home.

9 Die für die Auswertung der Daten notwendige Genehmigung durch die Bundesagentur für Arbeit liegt vor.

10 Da es keine Hinweise auf bundeslandspezifische Unterschiede gibt, wurde sich aufgrund der Anbindung an das Projekt FORWARD für Rheinland-Pfalz entschieden.

11 Eine Übersicht über Hinweise zum Akkreditierungsverfahren ist unter https://www.arbeitsagentur.de/datei/dok_ba014848.pdf einsehbar.

12 Vgl. z. B. VHS Rhein-Pfalz-Kreis: https://www.vhs-rpk.de/infos-service/qualitaet-anerkennungen bzw. VHS Mainz, Hinweis „Bildungsgutschein“: https://www.vhs-mainz.de/info-und-service/foerdermoglichkeiten/.

13 Vgl. § 16 Abs. 2 29. Corona-Bekämpfungsverordnung Rheinland-Pfalz (29. CoBeLVO) vom 03.12.2021: https://corona.rlp.de/fileadmin/corona/211203_29_CoBeLVO.pdf.

14 Vgl. hierzu die Empfehlungen zur Zulassung von Trägern und Maßnahmen des Beirats bei der Bundesagentur für Arbeit ab S. 12: https://www.arbeitsagentur.de/datei/dok_ba014848.pdf.
